# Developmental and Functional Hair Cell-Like Cells Induced by Atoh1 Overexpression in the Adult Mammalian Cochlea *In Vitro*

**DOI:** 10.1155/2020/8885813

**Published:** 2020-11-05

**Authors:** Lingyi Kong, Yuan Xin, Fanglu Chi, Jie Chen, Juanmei Yang

**Affiliations:** ^1^Department of Otolaryngology, Shanghai Children's Medical Center, Shanghai JiaoTong University School of Medicine, Shanghai, China; ^2^Department of Otology and Skull Base Surgery, Eye Ear Nose and Throat Hospital, Fudan University, Shanghai, China; ^3^Shanghai Clinical Medical Center of Hearing Medicine, Shanghai, China; ^4^Key Laboratory of Hearing Medicine of National Health Commission of the People's Republic of China, Shanghai, China; ^5^Research Institute of Otolaryngology, Fudan University, Shanghai, China

## Abstract

Hair cells (HCs) in the mammalian cochleae cannot spontaneously regenerate once damaged, resulting in permanent hearing loss. It has been shown that Atoh1 overexpression induces hair cell-like cells (HCLCs) in the cochlea of newborn rodents, but this is hard to achieve in adult mammals. In this study, we used a three-dimensional cochlear culture system and an adenoviral-mediated delivery vector to overexpress Atoh1 in adult mouse cochleae. HCLCs were successfully induced from 3 days after virus infection (3 DVI) *in vitro,* and the number increased with time. HCLCs were myosin7a positive and distinguishable from remnant HCs in a culture environment. Meanwhile, patch-clamp results showed that noninactive outward potassium currents (sustained outward potassium currents) could be recorded in HCLCs and that their magnitude increased with time, similar to normal HCs. Furthermore, transient HCN currents were recorded in some HCLCs, indicating that the HCLCs experienced a developmental stage similar to normal HCs. We also compared the electrophysiological features of HCLCs from adult mice with native HCs and found the HCLCs gradually matured, similar to the normal HCs. Meanwhile, HCLCs from adult mice possessed the same bundles as developmental HCs. However, these HCLCs did not express prestin, which is a special marker for outer hair cells (OHCs), even at 13 DVI. These results demonstrate that Atoh1 overexpression induces HCLC formation in the adult mammalian cochlea and that these HCLCs were functional and experienced a developmental process similar to that of normal HCs.

## 1. Introduction

Hair cells (HCs), which serve as inner ear sensory cells, are essential for the transduction of mechanical stimulation into hearing and balance signals in the inner ear. In lower vertebrates, such as birds and zebrafish, lost HCs can be replaced by spontaneous HC regeneration [1–4]. However, unlike these vertebrates, following acoustic overstimulation, ototoxic drug, trauma, or aging, HC loss in the mammalian inner ear is irreversible and is the primary reason for permanent sensorineural hearing loss [5–8].

HC reproduction in mammals has made great process in recent years, including the intervention of stem cells, supporting cell (SC) reprogramming, and the direct conversion of nonsensory epithelial cells of the cochlea [9–16]. Atoh1 is a basic helix-loop-helix transcription factor that is both necessary and sufficient for the development and genesis of HCs [17]. Atoh1 is gradually upregulated before HC formation and is downregulated during the HC maturation process [17–19]. The absence of Atoh1 in embryonic mice induces complete loss of both HCs and associated SCs [17, 19, 20]. In contrast, Atoh1 overexpression induces the formation of HCLCs both *in vitro* and *in vivo* [10, 21–23]. Meanwhile, it appears that HCLCs induced by different approaches remain in the developmental stage, which is a limiting factor for functional recovery [10, 22]. A recent study has shown that Atoh1 overexpression significantly enhanced HC regeneration and sustained functional recovery in the adult vestibule [11]. Meanwhile, another recent study also showed that multiple reprogramming, including Atoh1 overexpression, induces HCLCs in the adult cochlea *in vitro* and *in vivo* [24]. However, whether HCLCs induced by Atoh1 possess similar electrical physiological function as normal HCs is still unknown.

To address these questions, a human adenovirus serotype 5 (*Ad5*) vector encoding Atoh1 (*Atoh1*) and/or RFP (*RFP-Atoh1-*) or RFP-Atoh1 (*RFP-Atoh1+*) was used to overexpress Atoh1 in cultured mouse cochlea *in vitro*, and we assessed the morphological and functional features of HCLCs at different days after viral infection (DVI). Patch-clamp results showed the similarities and differences with respect to electrophysiological features between HCLCs and native HCs at different time points.

## 2. Material and Methods

### 2.1. Ethics Statement

This study was performed in accordance with the guidelines of the appropriate Institutional Animal Care and Use Committee of the Eye & ENT Hospital of Fudan University, Shanghai, China, and the Shanghai Children's Medical Center of Shanghai JiaoTong University School of Medicine, Shanghai, China.

### 2.2. Cochlear Explant Cultures and Viral Infection

**Figure 1:**
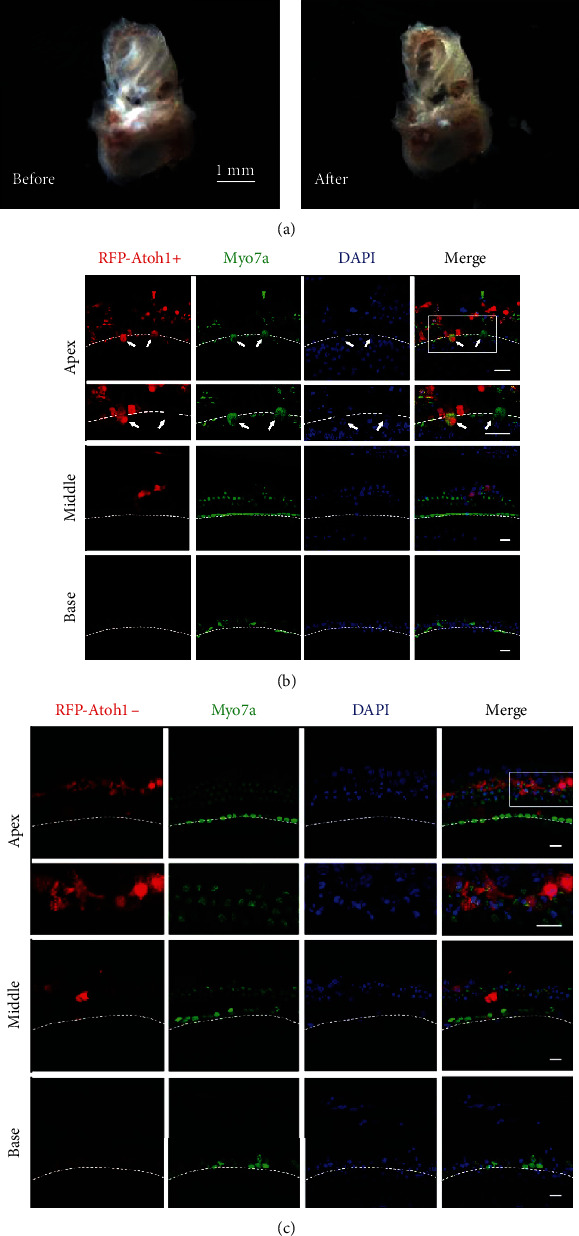
Atoh1 overexpression induces HCLC formation in the apical turn of adult mouse cochleae. (a) Establishment of adult cochlea explant cultures. A freshly dissected adult mouse cochlea with the intact bone (before) and with the holes perforated (after) before culture. (b, c) Infection status of *RFP-Atoh1+* in apical (apex), middle (middle), and basal (base) turns of mouse cochleae at 3 DVI. HCLCs (arrows) were found in the IHC region of apical turn. Remnant myosin7a+ HCs appear shrunken and in a dying state. *RFP-Atoh1-* were used as controls. Scale bars: 1 mm in (a), 15 *μ*m in (b) and (c). Rectangular boxes show areas from which high magnification and dashed lines mark the IHC region.

Adult C57BL/6 mice (JSJ Company, Shanghai, China), ranging from 8 to 10 weeks of age, were used in this study. Mice were decapitated, and the cochleae were carefully dissected out. Then, under a dissecting microscope (Zeiss), a hole was perforated at the apex of the cochlea, and the round window was opened and expanded by gently rotating the tip using a syringe needle. Using a fine-tipped pipette, phosphate buffer solution (PBS, HyClone) was gently flushed through the oval and round windows (as inlets) and the hole at the apex (as an outlet) (Figure 1(a)). Finally, the whole cochlea with the sensory epithelium was cultured in this experiment in Dulbecco's modified Eagle medium as follows: nutrient mixture F-12 (DMEM/F12, Invitrogen) medium containing 10% fetal bovine serum (FBS, Invitrogen) was used on the first day of culture.

The human adenovirus serotype 5 (*Ad5*) vector (with a final concentration of 1.0 × 10^8^ pfu/ml) encoding RFP and/or Atoh1 (*RFP-Atoh1+/-*) was used in this study. On the next day of culture, the virus diluted in the serum-free culture medium supplemented with B27 (Invitrogen) was added for a 12-hour infection and then replaced with serum-free medium every two days until the end of the culture. Petri dishes were kept in an incubator at 37°C with 5% CO_2_ during culture. The moment of adenovirus addition was recorded as day 0, then 1 day after viral infection (1 DVI), and so on.

### 2.3. Tissue Preparation and Immunofluorescence Staining

After 3 and 7 DVI, specimens were fixed in 4% paraformaldehyde (in PBS) at room temperature (RT) for 30 min followed by 10% ethylenediaminetetraacetic acid (EDTA) at RT for 8 hours to decalcify the bones. The bony capsule of the cochlea was dissected under the microscope. Then, the basilar membrane and associated organ of the Corti were carefully stripped from the modiolus. Next, the tectorial membrane and stria vascularis were torn off. The apical turn of the basilar membrane was prepared for immunofluorescence staining.

All specimens were blocked in 5% goat serum for 30 minutes at RT. Primary antibodies against myosin7a (1 : 200, mouse, DSHB, MYO7A 138-1) and/or prestin (1 : 200, Rabbit, Santa Cruz, sc-293212) diluted in 5% goat serum/0.1% Triton X-100/PBS were added overnight at 4°C. The next day, appropriate secondary antibodies, complementary to the primary antibodies, were used at RT for 2-3 hours in darkness. Secondary antibodies included donkey anti-rabbit Alexa Fluor 488 IgG (1 : 1000, Jackson, 112-546-143) and/or donkey anti-mouse Alexa Fluor 555 IgG (1 : 1000, Jackson,715-165-151). Bundles were detected by Alexa Fluor 488-labeled phalloidin (1 : 1000, Thermo Fisher, A12379). 4′,6-diamidino-2-phenylindole (DAPI; 1 : 1000, Sigma, D9542), stained for 10 min, was used to visualize nuclei. Slides were mounted and placed in a slide box at 4°C overnight to dry. Subsequently, slides were visualized under a Zeiss LSM 510 confocal laser-scanning microscope (Carl Zeiss, Germany).

### 2.4. Electrophysiological Recordings

Whole-cell patch-clamp recordings (HEKA, Germany) were used to detect electrophysiological properties of specimens (the apical turn of the basilar membrane) on 3, 5, and 7-9 DVI. Extracellular solutions (mM) are summarized as follows: 144 NaCl, 5.8 KCl, 0.9 MgCl2, 1.3 CaCl2, 10 HEPES, 5.6 glucose, pH 7.2-7.3, and osmotic pressure: 300-310 mOsm. Patch pipette filling solutions contained (mM) 1 EGTA, 135 KCl, 2 MgCl2, 0.1 CaCl2, 5 Mg2ATP, 0.5 NaGTP, 10 HEPES, 10 Na_2_ phoshocreatine, pH 7.2-7.3, and osmotic pressure: 290-300 mOsm. Borosilicate glass capillaries (OD 1.5 mm, ID 1.0 mm) were used to prepare patch pipettes on a horizontal multistep puller (Sutter con. USA). In the bath, pipettes had a resistance of 6-8 M*Ω*. Seal resistances higher than 1 M*Ω* were considered for recording. Currents were amplified using an Axopatch 200B amplifier (Molecular Devices, Union City, CA, USA), and data were digitized using an analog-to-digital converter (Digidata 1322; Molecular Devices, USA). Currents with a leak current less than 100 pA were accepted for statistical analysis.

### 2.5. Statistical Analysis

Statistical analyses were performed using GraphPad Prism (v8.0c software, GraphPad, San Diego, CA, USA). Statistical significance was determined using Student's *t*-tests and two-way ANOVA followed by Bonferroni's multiple comparisons test. Electrophysiological data were analyzed using Igor (HEKA Instruments, USA) and Origin Pro 8.0 (Microcal Software, USA). Nonlinear regression analysis was performed to quantify the goodness-of-fit via computation of the Boltzmann equation. In this study, *n* values represent the number of biologically independent samples. Data are shown as the mean ± s.d., and *p* values less than 0.05 were considered significant: ^∗^*p* < 0.05, ^∗∗^*p* < 0.01, and ^∗∗∗^*p* < 0.001.

## 3. Results

### 3.1. Atoh1 Overexpression Induces HCLC Formation in the Sensory Epithelial Region in the Apical Turn of Adult Mouse Cochleae

As shown in Figures 1(a)–1(c), myosin7a+ HCLCs were detected in the sensory region in the Atoh1 overexpression group (*RFP-Atoh1+*) from 3 DVI at the apical turn. HCLCs were distinguishable from the remnant native HCs. In the control group, myosin7a+ native HCs were shrunken and relatively well arranged at 3 DVI (Figure 1(c)). In the Atoh1 overexpression group, myosin7a+ HCLCs (arrows) were much larger and rounder than the remnant native HCs (Figure 1(b)). At the middle and basal turns, the viral infection rate was low, and only a small amount of SCs was RFP positive with no HCLCs detected (Figures 1(b) and 1(c)).

**Figure 2:**
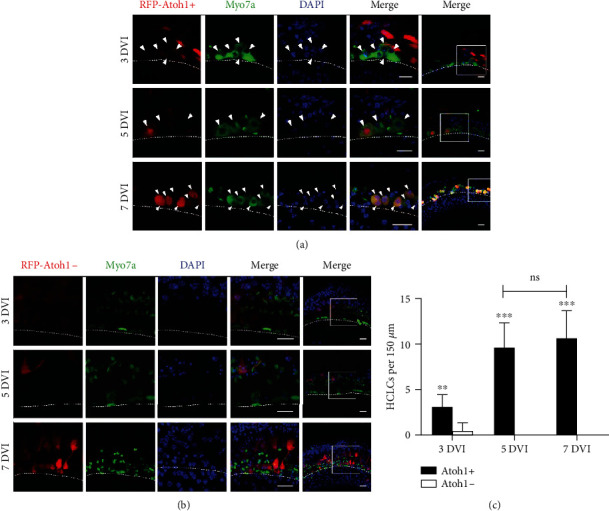
HCLCs were successfully induced from 3 DVI *in vitro*, and their numbers increased with time. (a, b) HCLCs (arrowheads) at 3, 5, and 7 DVI. *RFP-Atoh1-* was used as controls. (c) Quantification of HCLCs per 150 *μ*m cochlea length at 3, 5, and 7 DVI in both *Atoh1+* and *Atoh1-* groups (*n* = 5). Scale bars: 15 *μ*m in (a) and (b). Rectangular boxes show areas from which high magnification and dashed lines mark the IHC region.

At 5 and 7 DVI, myosin7a+ native HCs were also shrunken and scattered in the control group (Figure 2(b)). In the Atoh1 overexpression group, myosin7a+ HCLCs (arrowheads) were round or oval in shape and much larger than the native HC debris (Figure 2(a)). The number of HCLCs increased from 3.00 ± 1.41 at 3 DVI to 9.60 ± 2.70 at 5 DVI per 150 *μ*m along the long axis of the cochlea (*n* = 5), which is significantly higher than respective control levels. However, 7 DVI (10.60 ± 3.05; *n* = 5) was not significantly different from 5 DVI (Figure 2(c)).

### 3.2. Atoh1-Induced HCLCs Possess Bundles Similar to Developmental HCs but Do Not Express Prestin in Mature Cochleae

**Figure 3:**
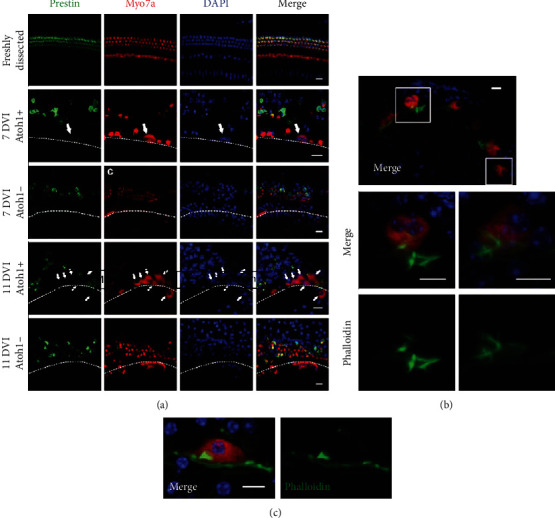
*Atoh1*-induced HCLCs possessed bundles similar to developmental HCs but did not express prestin in mature cochleae. (a) Distribution of prestin in a freshly dissected cochlea of an adult mouse. HCLCs (arrows) and remnant IHCs were prestin negative, and remnant OHCs were prestin positive at 7 and 11 DVI after *Atoh1* infection. (b) Hair cell bundles were observed at the apical side of HCLCs. (c) Several HCLC bundles were relatively well organized with V-shaped structures. Scale bar = 15 *μ*m in (a), 10 *μ*m in (b) and (c). Rectangular boxes show areas from which high magnification and dashed lines mark the IHC region.

Phalloidin was stained to detect whether HCLCs possess similar bundles as developmental HCs. According to our results, HC bundles were observed at the apical side of HCLCs (Figures 3(b) and 3(c)). Moreover, we used prestin, a specific motor protein expressed in the OHCs, to identify HCLCs at 7 and 11 DVI. As shown in Figure 3(a), although some shrunken OHC debris still expressed prestin with pyknotic nuclei, HCLCs were prestin negative both at 7 (*n* = 5) and 11 DVI (*n* = 3). In the control group, there were no HCLCs and only some scattered HC debris was prestin positive (*n* = 5) (Figure 3(a)).

### 3.3. Outward K+ and HCN Currents of Atoh1-Induced HCLCs in Mature Cochleae

A whole-cell patch-clamp recording technique was used to assess electrophysiological properties of HCLCs. Distinguishable among the HCLCs were remnant native HCs and nonsensory epithelial cells in the HC region. For native IHCs, we could see V-shaped bundles in a relative orderly arrangement. However, in native OHCs, the condition was too deteriorated to patch successfully (data not shown). For native IHCs, the condition was better than in native OHCs. For HCLCs, which were round and large, shown in red fluorescence and in better condition, they exhibited more durability in extracellular solutions during the patch time. Nonsensory epithelial cells in the sensory epithelium are finger shaped.

**Figure 4:**
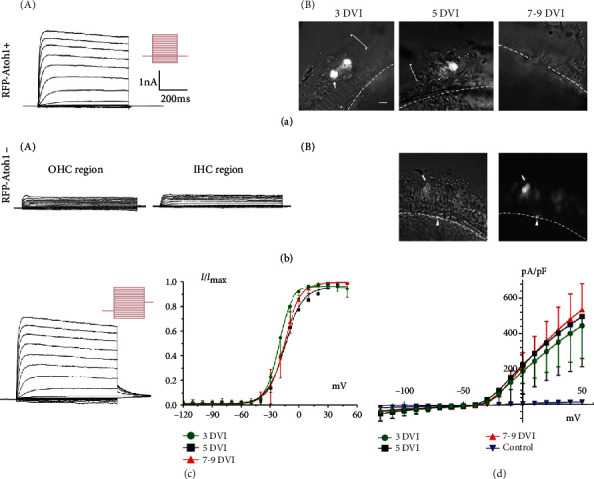
Sustained outward potassium currents of Atoh1-induced HCLCs in adult cochleae. (a) Representative samples of recorded sustained outward K+ currents (A) and micrographs of patched HCLCs (B) obtained from the 3, 5, and 7-9 DVI in the *Atoh1+* group. HCLCs are shown in RFP fluorescence and appear fresher. The voltage protocol is shown beside the traces. The cell clamp was held at a holding potential of -80 mv, and the voltage steps ranged from -120 to 50 mV in 10 mV increments to evoke voltage-dependent currents. (b) Representative outward K+ currents (A) and micrographs (B) of RFP+ cells (nonsensory epithelial cells) from the OHC and IHC regions in the control group. (c) Sustained outward K+ currents with tail currents and voltage-dependent activation curves of HCLCs. (d) I-V curves summarizing the magnitude of outward K+ currents in HCLCs from 3, 5, and 7-9 DVI and the control group, respectively. Scale bar = 20 *μ*m.

Sustained outward potassium (K+) currents in HCLCs were recorded in the *RFP-Atoh1+* group at 3, 5, and 7-9 DVI, which were 3.23 ± 1.42 nA (*n* = 9), 3.46 ± 0.14 nA (*n* = 5), and 3.89 ± 1.50 nA (*n* = 9), respectively (Figure 4(a)). In the *RFP-Atoh1-* (control) group, RFP+ cells in the sensory epithelial region merely expressed small sustained K+ currents, which were 400 ± 29.37 pA (*n* = 5). The remnant IHCs also expressed small sustained K+ currents, which were 390 ± 18.37 pA (*n* = 5) (Figure 4(b)). For HCLCs, the size of current-density relationships made no difference among the three *Atoh1+* groups, including 3, 5, and 7-9 DVI; however, all three groups were significantly larger than the control group (*p* < 0.01) (Figure 4(d)). Activation curves were obtained by analyzing tail currents at a fixed membrane potential (-30 mV), and voltage-dependent activation curves at 3, 5, and 7-9 DVI were independently fitted with the Boltzmann equation. The results showed significant differences (*p* < 0.01) (Figure 4(c)).

**Figure 5:**
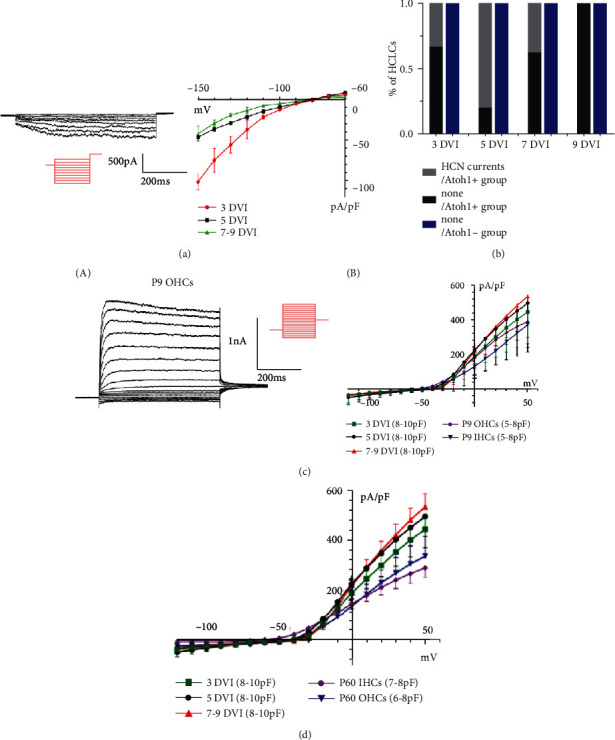
HCN currents of Atoh1-induced HCLCs and comparison of outward potassium currents of HCLCs from adult mice to normal HCs. (a) Representative HCN currents and I-V curves of HCLCs. The cell clamp was held at a holding potential of -70 mv, and the voltage steps ranged from -150 to -60 mV in 10 mV increments to evoke voltage-dependent currents. (b) Bar graph demonstrating the proportion of recorded HCN currents. (c) Representative outward K+ currents (A) recorded from P9 OHC. The I-V curves (B) show amplitudes of current density of OHCs and IHCs in P9 cochlea and HCLCs induced by Atoh1 in adult cochleae. (d) The I-V curves of OHCs and IHCs in P60 cochleae and Atoh1-induced HCLCs in adult cochleae.

Figure 5(a) shows 7HCN currents, as time-dependent inward rectifier K+/Na+ permeable conductance, recorded at different time points, which were 1.59 ± 0.96 nA (*n* = 3), 1.11 ± 0.55 nA (*n* = 4), and 0.84 ± 0.36 nA (*n* = 3). The amplitude of current density gradually decreased and then disappeared in sequence from 3 to 9 DVI, and differences were statistically significant (*p* < 0.01). Meanwhile, the proportion of recorded HCN currents primitively increased from 33.3% (3/9) at 3 DVI to 80% at 5 DVI (4/5), whereas it decreased to 37.5% on 7 DVI (3/8), suggesting that the appearance of HCN currents was a transient change (Figure 5(b)). By 9 DVI, HCN currents could no longer be recorded (0/5).

### 3.4. Comparison of Electrophysiological Properties of HCLCs from Adult Mice with Normal HCs

To compare electrophysiological properties of HCLCs and normal HCs, outward K+ currents of HCLCs and OHCs in the developmental stage were measured. Although recorded currents from these cells seemed similar to HCLCs, the mean membrane capacitance (Cm) was different, as shown in Figures 5(c) and 5(d). First, the sustained outward K+ currents of the inner and outer HCs of wild-type P9 mice were recorded, which were 2.09 ± 0.57 nA (*n* = 5) and 1.84 ± 0.21 nA (*n* = 5), respectively (Figure 5(c)). On 3 DVI, the mean amplitude of the current density of HCLCs showed no statistical difference from the inner and outer HCs. Another two groups (5 and 7-9 DVI) were higher than IHCs in P9 mice (*p* < 0.05); however, they still showed no statistical significant compared to OHCs. Second, the K+ currents of the inner and outer HCs in adult (P60) cochleae were also recorded, which were 2.73 ± 0.64 nA (*n* = 5) and 2.24 ± 0.28 nA (*n* = 5), respectively (Figure 5(d)). The mean amplitude of current density in the three groups (3, 5, and 7-9 DVI) was higher than the HCs in the adult cochlea (*p* < 0.01).

## 4. Discussion

In recent years, great progress has been made in HC regeneration in neonatal and adult cochleae [10, 11, 16, 24–27]. Recent studies have shown that sustained Atoh1 overexpression enhances the maturation of regenerated HCs in the damaged vestibular system [11, 12, 28]. HCs induced by Atoh1 in neonatal mammals are reminiscent of “ancestral” HCs that have commonalities with vestibular HCs [11, 29]. However, initial Atoh1 overexpression levels are correlative with mature levels and arrangement of HCLCs [30]. Higher initial Atoh1 overexpression levels induce more mature HCLCs according to electrophysiological results [30].

In this study, Atoh1 overexpression induced HCLCs in adult mice *in vitro*. On 3 DVI, several HCLCs were myosin7a positive in the sensory region, especially in the IHC region. Some HCLCs overlapped with RFP fluorescence, and some did not, consistent with previous studies [30, 31], due to Atoh1 expression level decreasing once HCLCs become mature. Meanwhile, HCLCs were distinguishable from remnant HCs. HCLCs exhibited a much rounder, bigger, and fresher appearance. Remnant HCs seemed to shrink and be in a dying state. The number of HCLCs increased from 3 DVI to 7 DVI. HCLCs at 7 DVI still failed to express prestin, a marker of mature OHCs. This is likely because the HCLCs were not mature enough, or perhaps the HCLCs were IHC-like cells. The HCLCs possessed bundles at the apical aspect similar to the developmental HCs, indicating incomplete maturity.

Compared to ectopic HCLCs induced from the neonatal inner ear, HCLCs from adult mice were primarily in the sensory region, especially the IHC region. Ectopic HCLCs induced from the neonatal inner ear are primarily from the nonsensory region, including the LER (lesser epithelial region) and GER (greater epithelial region) [29, 31–33]. This is mostly due to the LER and GER cells being stretched in the culture environment, which facilitates viral infection. For the adult cochlear culture, we reserved the cochlear bone, and the sensory region was the infection area. The IHC region in adult sensory epithelium presents a relatively loose structure, facilitating adenovirus infection. In the control group, no HCLCs were detected. Remnant HCs died in the culture environment, and cell debris was also myosin7a positive and even prestin positive. HCLCs *in situ* may directly build connections with the tectorial membrane and associated neurons, which may lead to eliciting mechanical transduction for hearing restoration.

In the cochlea, Atoh1-induced HCLC formation occurs primarily through direct transdifferentiation due to the limitations of the proliferation of SCs [34]. In our study, there were no HCLCs detected in the control group, indicating HCLC formation depends on Atoh1 overexpression, and there was no HC regeneration in the adult cochlea, distinct from the adult vestibule.

From embryonic development to maturation, HCs undergo an extremely complicated maturation process [7, 29, 35–38]. In addition to a series of related protein expression, the expression of outward K+ and HCN currents in HCs also goes through developmental changes. In the developmental stage, cochlear HCs express nondeactivated outward K+ current (sustained outward K+ current) and do not express fast deactivated outward K+ current (transient outward K+ current), and the amplitude increases with the time as HCs mature [35, 37, 39, 40]. Meanwhile, cochlear OHCs and IHCs transiently express HCN currents [36, 39].

A previous study reported that after Atoh1 overexpression, a fast deactivated outward potassium rectifier (*I*_K_) first appeared in most of the ectopic HCs, followed by sustained outward K+ current appearing later, and the proportion of cells with the sustained current increased over time as the HCLCs matured [29]. This means that HCLCs with no deactivated outward K+ current are more mature than HCLCs with fast-deactivated outward potassium. Similarly, HCN current first appeared in most of the ectopic HCs, then disappeared, and the proportion of cells with HCN current decreased over time as the HCLCs matured [29], indicating that HCLCs with HCN current are more immature. Thus, these ectopic HCLCs seem primordial. Another study reported that initial Atoh1 overexpression levels define the maturity level and arrangement of HCLCs, with higher initial Atoh1 overexpression levels inducing more mature HCLCs according to electrophysiological results, which process the higher proportion of sustained outward K+ current and lower proportion of HCN current [25].

For HCLCs induced by Atoh1 in adult cochlea, no transient outward current was recorded, and all of the HCLCs expressed sustained outward K+ current, distinct from ectopic HCLCs harvested from neonatal cochlea. These results may suggest that HCLCs from adult cochlea induced by Atoh1 exhibit faster development. HCLCs from adult cochlea also transiently expressed HCN currents, indicating that HCLCs from adult cochlea also experienced a developmental process.

We also compared HCLCs from adult cochleae at different time points with normal inner and outer HCs at P9 and P60. For mouse IHCs, a very small outward K+ current debuts at E14.5. Later, spontaneous action potentials (SAPs) occur at E17.5, and the magnitude of outward K+ currents increases steadily until the onset of hearing (about P12-14) [35, 37, 41]. Therefore, the magnitude of outward K+ currents in IHCs of P9 cochlea is on the rise. Judging from our research results, the maturity of HCLCs on 3 DVI was similar to IHCs in P9 cochlea. However, at 5 and 7-9 DVI, the HCLCs from adult cochlea were more mature than IHCs in the P9 cochlea (Figure 5(c)). Maturation of OHCs is achieved at about P8, a little earlier than in IHCs. In our study, the magnitudes of outward K+ currents in HCLCs at all time points were similar to normal OHCs in P9 cochlea. Furthermore, HCLCs from adult cochlea expressed sustained outward K+ current without the transient outward currents observed in native HCs. In general, these results demonstrated that outward K+ currents in HCLCs process a developmental regularity and synchrony, the same as normal HCs.

HCN currents, also known as inward rectifier (Ih) currents, have been largely recorded in immature HCs and regenerated ectopic HCs [36, 38, 41]. Similar to IK1 current in developing HCs, the function of the HCN current is setting the resting potential and regulating potential repolarization [36]. The inwardly transient currents will disappear abruptly when the HCs reach maturity, between approximately P12 and P14 in IHCs and earlier in OHCs [35, 36]. In our study, transient expression of HCN currents from 3 to 9 DVI mimicked the developmental process as that found in normal HCs. Additionally, the time point that HCN currents disappeared was on 9 DVI, about one week earlier than normal HCs and ectopic HCLCs induced from the neonatal inner ear, strongly indicating that functional development of the HCLCs was occurring relatively faster.

The cell state of remnant IHCs and OHCs degenerated in the culture environment. For remnant IHCs in the culture, only small sustained outward current could be recorded. For the OHC remnants in the culture environment, we were unable to even patch the cells.

## 5. Conclusions

In this study, HCLCs were induced in adult mouse cochleae by Atoh1 overexpression, and they were distinguishable in the sensory region. HCLCs expressed sustained outward K+ currents, and the magnitude increased with time similar to the developmental HCs. They also transiently expressed HCN channel currents similar to HCs, mimicking the developing stage. Meanwhile, HC bundles were detected in HCLCs in a developmental process, but they did not express prestin. These findings indicate that HCLCs from adult mice induced by Atoh1 overexpression undergo a similar developmental process as that of normal HCs, thereby providing guideline for future research into hearing restoration.

## Data Availability

All data used to support the findings of this study are available from the corresponding author upon request.
